# Conventional androgen deprivation therapy is associated with an increased risk of fracture in advanced prostate cancer, a nationwide population-based study

**DOI:** 10.1371/journal.pone.0279981

**Published:** 2023-01-04

**Authors:** Wei-Cheng Chen, Jian-Ri Li, Shian-Shiang Wang, Chuan-Shu Chen, Chen-Li Cheng, Sheng-Chun Hung, Ching-Heng Lin, Kun-Yuan Chiu, Po-Chi Liao

**Affiliations:** 1 Division of Urology, Department of Surgery, Taichung Veterans General Hospital, Taichung, Taiwan; 2 Division of Surgical Intensive Care Unit, Department of Intensive Care, Taichung Veterans General Hospital, Taichung, Taiwan; 3 Institute of Medicine, Chung Shan Medical University, Taichung, Taiwan; 4 Department of Medicine and Nursing, Hungkuang University, Taichung, Taiwan; 5 Institute of Biomedical Science, National Chung Hsing University, Taichung, Taiwan; 6 Department of Applied Chemistry, National Chi Nan University, Nantou, Taiwan; 7 Department of Post-Baccalaureate Medicine, College of Medicine, National Chung Hsing University, Taichung, Taiwan; 8 Department of Medical Research, Taichung Veterans General Hospital, Taichung, Taiwan; Chung Shan Medical University, TAIWAN

## Abstract

**Purpose:**

Androgen deprivation therapy (ADT) is the standard of care in advanced prostate cancer. We conducted a Taiwan National Health Insurance Research Database (NHIRD) study to evaluate the association between ADT and fracture risk in patient with prostate cancer in Taiwan.

**Methods:**

Between 2001 and 2008, data from the Taiwan NHIRD was collected. We separated newly diagnosed prostate cancer patients into four groups: the injection of gonadotropin-releasing hormone agonists and antagonists group, the orchiectomy group, the oral antiandorgens group and the radical prostatectomy only group. A non-cancer matched control group was also assigned for comparison. T tests, chi-squared tests, multivariate Cox proportional hazard regression were performed. A subsequent fracture event was defined according to the appropriate diagnosis codes (ICD9-CM 800–829) with hospitalization. Patients with fracture before their diagnosis with prostate cancer were excluded.

**Results:**

Overall, 22517 newly diagnosed patients with prostate cancer were enrolled in the study. After exclusion criteria were applied, 13321 patients were separated into the injection group (5020 subjects), the orchiectomy group (1193 subjects), the oral group (6059 subjects) and the radical prostatectomy only group (1049 subjects). The mean age of the overall study population was 74.4 years. Multi-variant analysis disclosed a significantly increased risk of fracture in the injection group, the orchiectomy group, and the oral group (hazard ratio [HR] = 1.55, 95%, confidence interval [CI] 1.36 to 1.76, p<0.001, HR = 1.95, 95%, CI 1.61 to 2.37, p<0.001, HR = 1.37, 95%, CI 1.22 to 1.53, p<0.001, respectively). In contrast, a significantly decreased fracture risk was noted in the radical prostatectomy only group (HR = 0.51, 95%, CI 0.35 to 0.74, p = 0.001). Patients receiving osteoporosis medication had a significantly decreased fracture risk (HR = 0.26, 95%, CI 0.19–0.37, p<0.001).

**Conclusions:**

ADT is associated with an increased risk of fracture. For patients receiving long-term prostate cancer castration therapy, doctors should always keep this complication in mind and arrange proper monitoring and provide timely osteoporosis medication.

## Introduction

Androgen deprivation therapy (ADT) is the standard of care for advanced prostate cancer and improved the survival of prostate cancer patients [1–3]. ADT includes the induction of hypogonadism through orchiectomy and a luteinizing hormone-releasing hormone (LH-RH) agonist, alone or combined with an androgen blockade (LH-RH agonist plus antiandrogen) [4,5] However, ADT decreases androgen activity, causing lower estrogen. In men, estrogen is produced by the aromatization of androgen, which regulates RANKL signaling pathway. A reduction in estrogen leads to lowered osteoblast activity and a subsequently knock down in trabecular bone mass. On the other hand, osteoclast number and activity were elevated, bringing about an increase in bone resorption, osteoporosis and fracture risk [6,7].

ADT also increased the risk of fall. Previous epidemiology studies showed that the increased risk of fall may have been due to the mental influences of ADT. An increased risk of dementia and/ or Alzheimer disease was noted in men receiving ADT for prostate cancer compared with men who did not, especially when the treatment interval was over 12 months, resulting in a heightened risk of fall. In both eastern and western studies, both risk factors increased the rate of fracture, further mortality, and morbidity [8–11].

Several other factors could also be associated with the increased risk of fracture in prostate cancer patients, including age, advanced disease stage, the use of bone protective agents and comorbidity [12,13].

Herein, we conducted a study using the Taiwan NHIRD to understand whether ADT cause an increased fracture risk in prostate cancer patients in Taiwan, and whether osteoporosis treatment agents could counter any adverse effects.

## Materials and methods

We collected data from the Taiwan NHIRD between 2001 and 2008 according to the protocol, which was approved by the institutional review board of Taichung Veterans General Hospital (approved no. CE13151-1). Statistically, there were no significant differences in the age and healthcare provided between the study group and the general population of the NHIRD. Data on ambulatory care, inpatient care, as well as prescribed medications was collected from the patient medical records. All diagnoses were in accordance with the International Classification of Diseases, ninth revision (ICD-9).

The Taiwan government launched single-payer mandatory enrollmentin the National Health Insurance Program since March 1^st^ 1995, with all health data recorded in the Taiwan NHIRD. It covers more than 99% of the Taiwan population and access to the NHIRD for our study analysis was provided by the NHRI Ethics Review Committee.

### Study population and study endpoint

**Fig 1:**
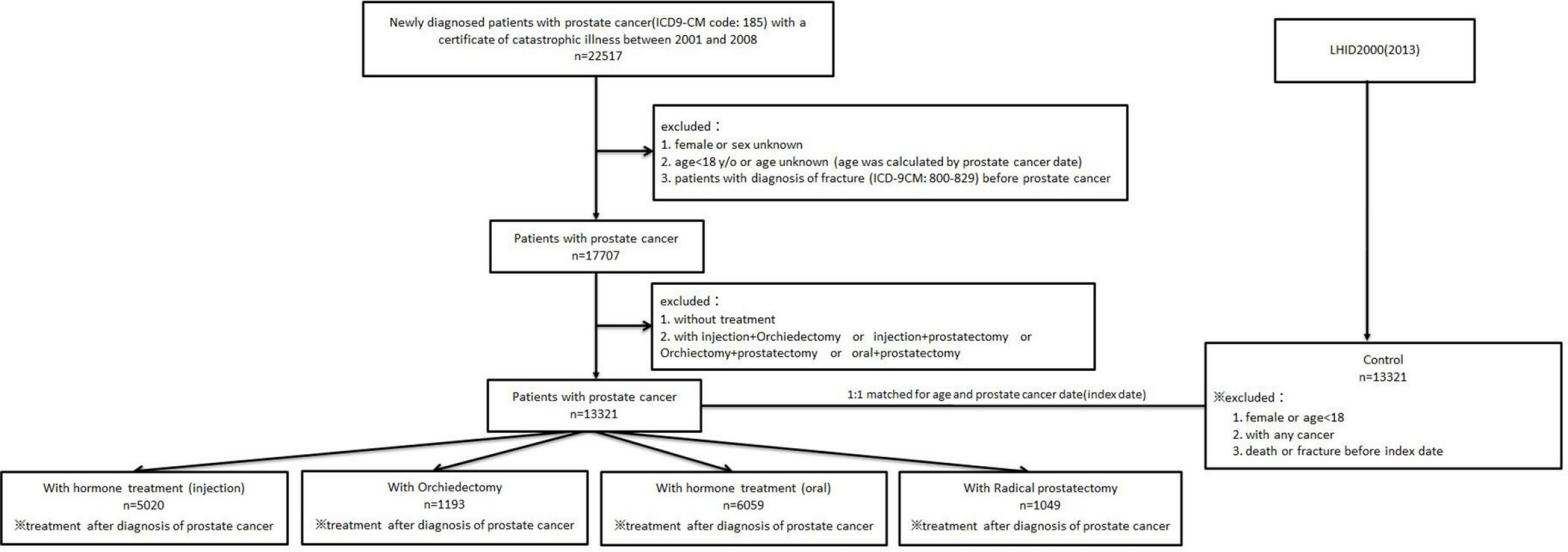
Flow diagram showing the process of fracture patient sampling and participation.

Overall, we gathered 4 groups of newly diagnosed prostate cancer patients. This included patients who received GnRH agonists or antagonists only (allowing 2 weeks for combination with anti-androgens), patients who received conventional anti-androgens only (excluding abiraterone acetate, enzalutamide, darolutamide, apalutamide and other new generation antiandrogen regimens), patients who received orchiectomy only, and patients who received radical prostatectomy only, as well as a group of non-cancer control patients for comparison. All study patients were newly diagnosed with prostate cancer between 2001 and 2008, and followed until 2013. Different types of ADT were classified as described above using the NHI drug code and surgery code. The study flow diagram in Fig 1 presents the patient collection and sampling principles. This study was conducted on the assumption that ADT confers bone fracture adverse events on prostate cancer patients. The primary study endpoint was a new event of bone fracture censored with patient admissions after the prostate cancer diagnosis date.

For patients to be included in the study groups they must meet the following criteria: (1) newly diagnosed with prostate cancer between 2001 and 2008; and (2) receiving definite therapy as defined in the corresponding group. Patients were excluded when they met one of the following criteria (1) receiving combined ADT for more than 2 weeks (2) received both a radical prostatectomy and ADT during their lifetime (3) concomitant presence of another cancer during the study period (4) preexisting bone fractures before the prostate cancer diagnosis. The diagnosis code for prostate cancer was ICD-9-CM 185.0. We also documented, myocardial infarction (ICD-9CM: 410,412,A270), heart failure (ICD-9CM: 428), peripheral vascular disease (ICD-9CM: 441, 4439, 7854, V434), stroke (ICD-9CM: 430–438, A290-A294, A299), dementia (ICD-9CM: 290, 331.0, 331.2, A210), pulmonary disease (ICD-9CM: 490, 491, 492, 493, 494, 495, 496, 500, 501, 502, 503, 504, 505), connective tissue disorder (ICD-9CM: 7100, 7101, 7104, 7140, 7141, 7142, 71481, 5171, 725), liver disease (ICD-9CM: 571, 572), Diabetes (ICD-9CM: 250, A181), paraplegia (ICD-9CM: 342, 3441) and chronic kidney disease (ICD-9CM: 403, 404, 582, 583, 585, 586, 588, V42.0, V45.1, V56, A350) as modified factors. A subsequent fracture event was defined according to the relevant diagnosis code (ICD9-CM 800–829) with hospitalization. Patients with fracture before their diagnosis of prostate cancer were excluded.

### Statistical analysis

For continuous variables, we presented the data as the mean ± standard deviation (SD). For categorical variables, we presented the data as proportions. A t test was used to analyze the differences between continuous variables, while a chi-squared test was used for categorical variables. Multivariate Cox proportional hazard regression was applied to determine the association between the prevalence of fracture among the divided groups to estimate the hazard ratio (HR) as well as the 95% confidence interval (CI). This association was further confirmed using propensity analysis. A log-rank test was applied to examine the statistical significance of the cumulative incidence curves, and then plotted using the Kaplan-Meier method. All statistical analyses mentioned above were performed using SAS software version 9.2 (SAS Institute Inc., Cary, NC, USA). Statistical significance was defined as a p value <0.05.

## Results

A total 22517 newly diagnosed patients with prostate cancer (ICD9-CM code: 185) with a certificate of catastrophic illness between 2001 and 2008 met the primary inclusion criteria.

After exclusion of patients with a female or unknown sex, aged <18 years or age unknown, with a diagnosis of fracture before their prostate cancer diagnosis, who went without treatment, who received injected hormone therapy and orchiectomy, injected hormone therapy and prostatectomy, orchiectomy and prostatectomy or oral hormone therapy and prostatectomy, a total of 13,321 cases were selected as the study subjects (Fig 1). Among them, 5020 subjects were included in the injection group, 1,193 subjects were included in the orchiectomy group, 6,059 subjects were included in the oral group and 1,049 subjects were included in the radical prostatectomy group. The control group also included 13,321 randomly selected subjects from a 1 million non-cancer population with 1:1 matching to the study group.

S1 File shows a comparison of the basic characteristics between the study groups and the healthy controls. The mean age of the total study patient cohorts was 74.3 years.

There were no significant differences between the study group and the control group regarding age, myocardial infarction, connective tissue disease and paraplegia. A comparison of comorbidities, it showed high heterogeneity between the control group and the different study groups including for heart failure, peripheral vascular disease, stroke, dementia, pulmonary disease, liver disease, diabetes, and renal disease. Fewer patients accepted osteoporosis medication in the control group compared with the study groups.

**Fig 2:**
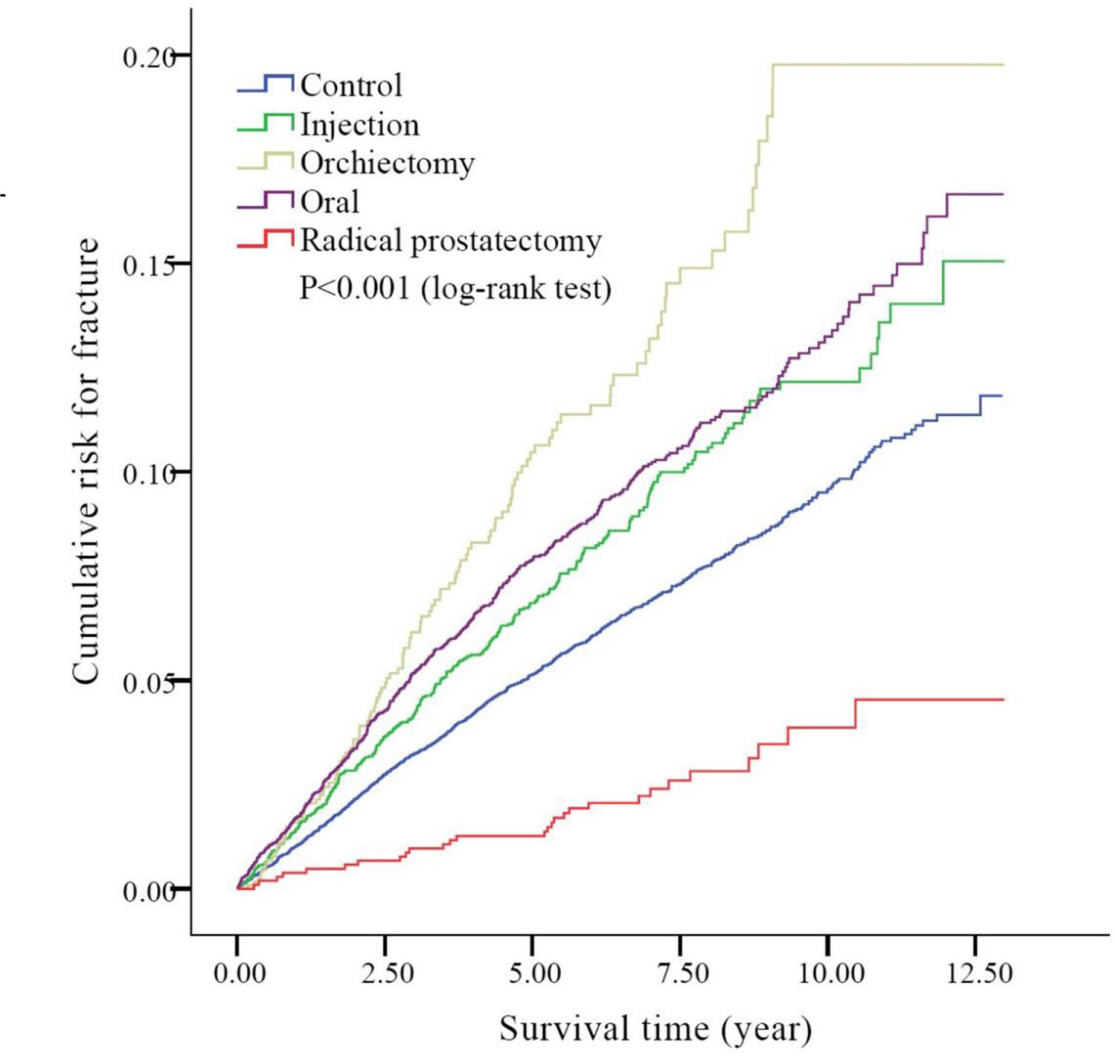
Kaplan-Meier cumulative incidence of developing fracture among five groups (P<0.001, log-rank test) (n = 26,642).

After adjusting for all variables (S2 File), a significantly increased risk of fracture was observed in the injection group, the orchiectomy group, and the oral group (HR = 1.55, 95% CI 1.36 to 1.76, p<0.001, HR = 1.95, 95% CI 1.61 to 2.37, p<0.001, HR = 1.37, 95% CI 1.22 to 1.53, p<0.001 respectively) but a lower risk of fracture was observed in the radical prostatectomy group compared with the control group (HR = 0.51, 95% CI 0.35 to 0.74, p = 0.001) (Fig 2).

Age (HR = 1.04, 95% CI 1.03 to 1.05, p<0.001) and comorbidity with heart failure (HR = 1.24, 95% CI 1.02 to 1.51, p = 0.034), stroke (HR = 1.15, 95% CI 1.01 to 1.31, p = 0.035), pulmonary disease (HR = 1.22, 95% CI 1.10 to 1.36, p<0.001) and renal disease (HR = 1.33, 95% CI 1.14 to 1.56, p<0.001) increased the risk of fracture in prostate cancer patients, while the use of osteoporosis medication significantly decreased the risk of fracture (HR = 0.26, 95% CI 0.19 to 0.37, p<0.001).

## Discussion

In our study, we found that the use of all kinds of ADT significantly increased the risk of bone fracture in the treatment of prostate cancer compared with the normal population.

Many previous studies have proved the relationship between the risk of bone fracture and the use of ADT in prostate cancer patients. A landmark study in 2005 found that 19.4 percent of those who received ADT had a fracture, compared with 12.6 percent of those not receiving ADT (p<0.001). ADT may increase the risk of fracture by 1.5-fold and the incidence of hospitalization due to fractures by 1.7-fold [14]. A population based study in a western community also showed that ADT was associated with an increased risk of fracture [12,15–19]. A recent Asian nationwide population study in Korea revealed similar results [20]. C.-T. Wu et al. reported that ADT increaseed the risk of fracture in a Taiwan population but this increase was seemingly not as large as that observed in Western populations [21].

To date, there has been no large study that compared how different ADT regimen were related to the risk of bone fracture. Although there was no statistically significant difference, we found that patients who received orchiectomy had the highest risk of fracture compared with those who accepted oral ADT or injected ADT. Wang A et al. reported that those who received combined androgen blockade (OR = 3.48; 95% CI 3.07–3.96) and bilateral orchiectomy with pharmacologic ADT (OR = 4.32; 95% CI 3.34–5.58) had the greatest risk of fracture, while a study from Lee CH et al. showed no significant difference in the relative risks among the three types of ADT [19,22]. One systematic review and meta-analysis reported that the use of androgen receptor inhibitors (ARIs) was associated with a 1.8 times higher risk of fall and a 1.6 times higher risk of fracture compared with a placebo and ADT group [23]. Novel ARIs, such as enzalutamide and abiraterone were not included in this study, as these ARIs were only approved for use from 2018 in Taiwan. A population based study of a more recent cohort may tell us more information about the comparative risk of bone fracture between ADTs in the era of ARIs.

Several comorbidities were reported to exacerbate the risk of bone fracture in ADT-targeted patients with prostate cancer, including diabetes, autoimmune disease, and liver disease [13,24]. However, we found that diabetes, connective tissue disorder, and liver disease had no significant association with an increased fracture risk in this group of patients. Instead, pulmonary disease, renal disease, heart failure, stroke and the use of osteoporosis medication significantly reduced the risk of fracture in men with prostate cancer.

Bone protective agents are recommended for the prevention of skeletal-related events (SRE) in patients with metastatic castration-resistant prostate cancer [7,25–28]. Treatment with bisphosphonates showed a significant effect at preventing fractures (risk ratio [RR], 0.80; p = 0.005) and osteoporosis (RR, 0.39; p <0.00001). (15, 16) Our study revealed that bone protective agents significantly lowered the risk of fracture among all groups of prostate cancer patients (HR = 0.26, 95% CI 0.96 to 0.37, p<0.001). Although the bone protective agents clodronate, zoledronic acid, denosumab, risedronate, alendronate and teriparatide were included in our study, there may have been very few patients who accepted treatment with denosumab as a bone protective agent, because denosumab was only approved for use in Taiwan by the FDA in 2013. Further population based studies are needed to compare the efficacy of novel denosumab treatment at lowering fracture risks compared with traditional bisphosphonate.

We also found that the risk of fracture was significantly lower in the OP only group compared with the ADT group and even in the normal population (Fig 2). Less comorbidities were found in the OP only group (S1 File), which indicated a healthier general condition and less pre-treatment risk factors for fracture. Metastatic disease to the bone is associated with SREs, including pathologic fractures and a risk of spinal cord compromise. Patients who undergo operations only mostly have a localized disease status. Localized disease status without the use of ADT and better general health maybe related to the lower risk of bone fracture.

There were several limitations of this study. First, clinical data such as tumor grade, stage, PSA or bone mineral density (BMD) were not presented. Tumor stage may be associated with the risk of fracture, such as bone metastasis instead of ADT related osteoporosis. Patients receiving ADT treatment were mostly at an advanced tumor stage such as metastatic disease while the OP only group may have primarily had relatively localized disease. Second, the ADT group, was only divided into three sub-groups, which were orchiectomy, injected and oral ADT, we didn’t further analyze the different types of ADT such as LHRH analog or antagonist. Third, pre-treatment BMD data was not collected so cause of preexisting osteoporosis may have been underestimated. With the use of denosumab and novel ARIs, a new population based cohort is necessary to provide a comparison of these different cohorts. Osteoarthritis is related to an increased incidence of fracture [29]. Patients with osteoporosis were at a higher risk of fracture [30]. We did not analyze the risk of fracture caused by osteoarthritis and osteoporosis, which was a limitation of our study. There is no evidence to date that ADT would cause osteoarthritis. Patients in the study group (with prostate cancer and ADT) and the healthy control group both shared the same risk of osteoarthritis and osteoporosis since we did not adjust for it.

## Conclusion

All types of ADT increased the risk of fracture compared with those patients who underwent prostatectomy only and the general population. Among the three groups, orchiectomy increased the fracture risk more than other types of ADT. Age and multiple comorbidities also increased the risk of fracture in prostate cancer patients, including heart failure, stroke, pulmonary disease and renal disease. The use of osteoporosis medication significantly decreased the risk of fracture events.

## Supporting information

S1 File (XLSX)

S2 File (XLSX)
